# Community violence in the HIV risk environment: a qualitative study among youth in Brazil

**DOI:** 10.1590/0102-311XEN041825

**Published:** 2026-03-23

**Authors:** Marina Katague, Daniela Knauth, Regina Barbosa, Luciana Teixeira, Andrea Fachel, Bruna Hentges, Jamila Stockman, Kiyomi Tsuyuki

**Affiliations:** 1 University of California San Diego, La Jolla, U.S.A.; 2 Universidade Federal do Rio Grande do Sul, Porto Alegre, Brasil.; 3 Universidade Estadual de Campinas, Campinas, Brasil.

**Keywords:** Exposure to Violence, HIV, Disease Prevention, Qualitative Research, Exposição à Violência, HIV, Prevenção de Doenças, Pesquisa Qualitativa, Exposición a la Violencia, VIH, Prevención de Enfermedades, Investigación Cualitativa

## Abstract

In Brazil, HIV infection rates are rising among youth aged 15-24 years. HIV prevention efforts often overlook social determinants of health as barriers to engaging in health behaviors. This study investigates how community violence influences HIV risk among youth. We conducted qualitative analysis of semi structured interviews with youth (N = 53) from two urban peripheries in São Paulo and Porto Alegre, using the HIV risk environment framework to guide analysis. Our findings indicate that police violence and gang activity are pervasive in youth-frequented spaces, disrupting daily life and creating an environment of fear and intimidation, limiting freedom of movement. Violence accelerates social insecurities, particularly among young males and Black youth, who are disproportionately targeted. Substance use links community violence to HIV risk. Community violence hinders educational attainment and employment opportunities, perpetuating cycles of poverty and instability. Aggravated burglaries and economic strain force individuals into high-risk activities, further entrenching them in violence and increasing vulnerability to HIV. Policing practices, characterized by aggressive interventions, exacerbate the HIV risk environment by fostering distrust and driving community members towards gangs for protection. While free HIV testing and treatment are available, barriers such as community violence hinder access to care. This study affirms the need for interventions that address the root causes of violence to improve the HIV risk environment and health disparities among at-risk Brazilian youth.

## Abstract (pt)

No Brasil, as taxas de infecção por HIV estão aumentando entre jovens de 15-24 anos. Os esforços de prevenção frequentemente ignoram determinantes sociais da saúde como barreiras relacionadas a comportamentos de saúde. Este estudo investiga como a violência comunitária influencia o risco de HIV entre os jovens. Realizamos uma análise qualitativa secundária de entrevistas semiestruturadas com jovens (N = 53) de duas periferias urbanas em São Paulo e Porto Alegre, utilizando o arcabouço do ambiente de risco para HIV. Nossas descobertas indicam que a violência policial e a atividade de gangues são generalizadas em espaços frequentados por jovens, perturbando o dia a dia e criando um ambiente de medo e intimidação que limitam a liberdade de circulação. A violência acelera as inseguranças sociais, especialmente entre jovens do sexo masculino e jovens negros, que são alvos de maneira desproporcional. O uso de substâncias liga a violência comunitária ao risco de HIV. A violência comunitária dificulta a realização educacional e as oportunidades de emprego, perpetuando ciclos de pobreza e instabilidade. Assaltos e a pressão econômica forçam os jovens a realizarem atividades de alto risco, aprofundando-os ainda mais na violência e aumentando a vulnerabilidade ao HIV. As práticas policiais, caracterizadas por intervenções agressivas, agravam o ambiente de risco de HIV ao fomentar a desconfiança e impulsionar membros da comunidade a se aproximarem de gangues em busca de proteção. Embora testes e tratamentos gratuitos para HIV estejam disponíveis, barreiras como a violência comunitária dificultam o acesso ao atendimento. Este estudo reforça a necessidade de intervenções que abordem as causas profundas da violência para melhorar o ambiente de risco de HIV e as disparidades de saúde entre jovens brasileiros em situação de risco.

## Abstract (es)

En Brasil, las tasas de infección por VIH están aumentando entre los jóvenes de 15-24 años. Las iniciativas de prevención del VIH suelen pasarporalto los determinantes social es de la salud como obstáculos para adoptar comportamientos saludables. Este estudio investiga cómo la violencia comunitaria influye en el riesgo de VIH entre los jóvenes. Realizamos un análisis cualitativo secundario de entrevistas semiestructuradas con jóvenes (N = 53) de dos periferias urbanas de São Paulo y Porto Alegre, utilizando el marco del entorno de riesgo de VIH. Los hallazgos indican que la violencia policial y la actividad de las pandillas son omnipresentes en los espacios frecuentados por los jóvenes, lo que perturba la vida cotidiana y crea un ambiente de miedo e intimidación, limitando la libertad de movimiento. La violencia acelera la inseguridad social, especialmente entre los jóvenes varones y los jóvenes negros, que son objeto de ataques de manera desproporcionada. El consumo de sustancias vincula la violencia comunitaria con el riesgo de contraer el VIH. La violencia comunitaria obstaculiza el rendimiento educativo y las oportunidades deempleo, perpetuando los ciclos de pobreza e inestabilidad. Los robos con agravantes y las dificultades económicas obligan a las personas a realizar actividades de alto riesgo, lo que las sumerge aún más en la violencia y aumenta su vulnerabilidad al VIH. Las prácticas policiales, caracterizadas por intervenciones agresivas, agravan el entorno de riesgo de VIH al fomentar la desconfianza y llevar a los miembros de la comunidad a buscar protección en las pandillas. Si bien se ofrecen pruebas y tratamientos gratuitos para el VIH, barreras como la violencia comunitaria impiden el acceso a la atención médica. Este estudio afirma la necesidad de intervenciones que aborden las causas fundamentales de la violencia para mejorar el entorno de riesgo de VIH y las disparidades en materia de salud entre los jóvenes brasileños en situación de riesgo.

## Introduction

Globally, youth account for 27% of all new HIV infections ^1^. In Brazil, the Ministry of Health reported rising HIV infection rates among people aged 15-24 years, despite overall declines in AIDS-related mortality ^2^. Limited prevention knowledge - often due to early school dropout before receiving sex education ^3^ - combined with high rates of condomless sex contributes to this trend ^4^ ^,^ ^5^. While adolescence is a critical formative stage for establishing health behaviors, effective HIV prevention should also address upstream determinants such as community violence.

The HIV risk environment framework highlights the role of sociostructural forces in shaping health behaviors, classifying environmental influences by type (physical, social, economic, policy) and level (micro, meso, macro) ^6^ ^,^ ^7^. This framework has been applied to examine how environmental factors, such as community violence and sex trafficking, influence HIV risk across diverse settings ^8^ ^,^ ^9^ ^,^ ^10^. However, the impacts of drug trafficking and police surveillance on the HIV risk environment remain underexplored in Brazilian contexts ^11^. 

Community violence, or exposure to public acts of violence ^12^, poses threats to public health. Law enforcement and gang violence have distinct health impacts for youth ^13^ ^,^ ^14^. Brazil’s police lethality rate, over two per 100,000 individuals, exceeds the total homicide rates of most Western European countries ^15^. Black Brazilians are nearly three times as likely to be killed by law enforcement than the general population ^16^. Law enforcement is often entangled with local drug markets controlled by criminal factions ^17^, and drug trafficking is connected to escalating community violence ^18^. Gang violence is also gendered, significantly increasing homicide rates among males aged 15-44 ^19^. Indirect effects of community violence include greater involvement in risky sex, elevated substance abuse, and more severe internalizing mental health symptoms ^14^ ^,^ ^20^ ^,^ ^21^. Witnessing violence also results in mental health needs ^22^, with post traumatic stress disorder commonly reported after exposure to community violence ^23^ ^,^ ^24^.

Research on how community violence influences HIV outcomes among youth remains limited. We address this gap by qualitatively exploring how community violence shapes the HIV risk environment among Brazilian youth. Our findings provide insight into how community violence, as a social determinant of health, influences HIV transmission.

## Methods

### Study design

Our team conducted an ethnographic study to understand the feasibility and acceptability of HIV self-testing for youth. We show a secondary analysis of the qualitative data generated, as the salience of community violence within participants’ narratives warranted a focused examination.

### Study setting

The study is situated in São Paulo (Brasilândia), State of São Paulo, and Porto Alegre (Restinga), State of Rio Grande do Sul, two urban peripheries with high HIV burden. São Paulo has the largest number of people living with HIV nationwide, whereas Porto Alegre has long reported the highest incidence rates of HIV and AIDS ^25^. Brasilândia has nearly 265,000 residents, high racial segregation, and one of the city’s highest youth homicide rates, with 40% of households earning up to two minimum wages ^26^. Restinga has more than 60,000 residents, nearly 40% identifying as Black or Mixed-race, high incidence of adolescent homicide, and a history as a resettlement zone that left chronic deficits in infrastructure and services ^26^ ^,^ ^27^ ^,^ ^28^. Both communities face chronic poverty and dense occupation, which sustain community violence.

### Data collection

We employed rapid assessment procedures (RAP) for formative research, combining different types of data collection ^29^. Recruitment and data collection occurred in 2019-2020. We conducted ethnographic observations and informal interviews with local leaders to map youth sociability and understand the role of youth-focused organizations. We used snowball sampling to reach participants. Youth respondents (n = 53) provided sociodemographic information, indicating their age, sex, skin color, and level of education. We then conducted semi-structured interviews focused on perceptions of HIV risk. 

### Ethics statement

The study received approval from the University of California San Diego Institutional Review Board (UCSD IRB #180832) and local IRB approval in Brazil from the IRBs at the State University of Campinas (UNICAMP, acronym in Portuguese; for São Paulo study) and the Federal University of Rio Grande do Sul (UFRGS, acronym in Portuguese; for Porto Alegre study).

### Data analysis

Interviews were transcribed verbatim, and the research team organized the text units into the framework of the interview guide in the context of the primary study. We coded the data using NVivo software (https://lumivero.com/products/nvivo/) ^30^. We then ran queries for all data involving violence, and interviews in Portuguese were translated. We conducted deductive content analysis to identify physical, social, economic, and policy factors within the HIV risk environment framework.

## Results

We conducted interviews with participants from São Paulo (n = 36) and Porto Alegre (n = 17). Table 1 summarizes participant demographics. The mean age of youth participants was 19.64 years (standard deviation, SD = 2.51) in São Paulo and 21.59 years (SD = 2.48) in Porto Alegre. São Paulo had predominantly female respondents (83%), whereas in Porto Alegre the distribution was more balanced (47% female, 53% male). Participants from São Paulo mostly identified as Black/Mixed-race (81%). In Porto Alegre, the distribution was 47% Black and 53% White. A total of 58% of São Paulo’s participants had less than a high school. In Porto Alegre, most (59%) had some higher education.

**Table 1:** Demographic characteristics of the participants of the interview.

Characteristics	São Paulo (n = 36)	Porto Alegre (n = 17)	Total (N = 53)
n (%)	n (%)	n (%)
Mean age (years) [SD]	19.64 ± 2.51	21.59 ± 2.48	20.37 ± 2.71
Sex			
Female	30 (83)	8 (47)	38 (72)
Male	6 (17)	9 (53)	15 (28)
Self-identified race/skin color			
Black	14 (39)	8 (47)	22 (42)
Mixed-race	15 (42)	0 (0)	15 (28)
White	7 (19)	9 (53)	16 (30)
Education level			
Lower than high school	21 (58)	5 (29)	26 (49)
High school	10 (28)	2 (12)	12 (23)
Incomplete higher education	5 (14)	10 (59)	15 (28)

SD: standard deviation.

Youth environments shape HIV risk behaviors and outcomes. Regarding HIV, one participant acknowledged, “Just by being young, being in the world, going out, dating, hooking up, you are already exposed to risk” (São Paulo). Boxes 1 and 2 show each risk environment type and related manifestations and direct participant quotes, respectively.

**Box 1:** Community violence in the HIV risk environment framework among youth in Brazil.

	MICRO-ENVIRONMENT	MACRO-ENVIRONMENT
Physical	Direct violence in youth-frequented spaces (bars/clubs, community centers, neighborhoods)	Drug trafficking and distribution routes in São Paulo and Porto Alegre
	Limited freedom of movement	Pervasive police surveillance
Social	Fear and intimidation inspiring avoidance behaviors	Systemic racism and marginalization of Black youth
	Stress, anxiety, and trauma	Gendered risk
Economic	Salience of robberies/aggravated burglaries	Scarcity of income generation/employment opportunities
	Interrupted school attendance	Growth of informal economies (drug trafficking, weapons trafficking)
Policy	Local access to HIV testing and care facilities	Ineffective laws governing protection of health and human rights fail to address the root causes of violence Inconsistent enforcement of laws governing drug possession and paraphernalia

**Box 2:** Representative quotations illustrating community violence within the HIV risk environment framework.

	REFERENCE	ILLUSTRATIVE QUOTE
Physical	Q1	“[The police] *arrive, they’re shooting, throwing gas bombs. Then people start running, then there is more confusion. I’ve run from them a lot, but it’s easier for us here in Community 1 to run, because here we already know everything. There are people that don’t know police catch and then beat them*” (São Paulo)
	Q2	“*You have to run. Some older boys, they had already drawn their guns to shoot the police. And we were all there in the middle of the dance party… running. They threw a bottle [back] at the police…The police came hitting, so they threw a bottle, they shot...*” (São Paulo)
	Q3	“*He had the rifle pointed at me, ‘hands on the wall, man!’, then I went to the wall, they kicked me, my brother, and little* [friend’s name] *in his legs. The kid was what, I think about 11, 10 years old? They were kicking the kid’s leg. I got pissed off attacking them, they put the rifle to my head like that, they kept pointing like ‘Where’s the drugs?’ And it’s just because I was drinking, you know? The woman police officer came just like that… she searched me, almost crushed our cell phones on the floor*” (Porto Alegre)
	Q4	“[A neighbor] *was telling me that her son was sitting on the sofa, and she was sweeping the house and they came in armed and killed him on the sofa. He was into drugs*” (São Paulo)
	Q5	“*We don’t go out on the street,” one participant shared, “because the trafficker gangs are currently at war, so my children haven’t been to class since the shootings started*” (Porto Alegre)
	Q6	“*They entered my courtyard and stole a lot of things, they stole my clothes from the clothesline, they stole my son's bicycle, they stole my father’s stuff. And my father went from drug dealer to drug dealer to find out if they had gone there to trade for drugs and he found out who did it. My father went out at dawn to look for the person and gave him a beating. He went and said to the robber’s mom, “I want everything back, otherwise I’m going to the police and I’m going to hand you over and your son. Then they had to give it back, and the robber didn’t even live for a long time. He stole from everyone, and then there came a time when someone put an end to him*” (Porto Alegre)
Social	Q7	“*I’m a little scared. Whenever I’m at a soccer game at the stadium, I feel that, as a Black person, I’m more vulnerable to violence*” (Porto Alegre)
	Q8	“*They* [(the police)] *go more because of drugs, there’s a lot of it... it also depends on the street*” (São Paulo)
	Q9	“*Nowadays I prefer to stay at home more than go out because the violence is too much, it’s very dangerous, we take it, we go out into the street and we don’t know, it’s not like... Before, it was always dangerous, but today it’s getting worse. I do stay at home*” (Porto Alegre)
Economic	Q10	“*He comes back* [from work] *with Uber because he was robbed in front of the bus stop near his workplace, because he leaves around midnight. He was robbed and they took everything from him; he had rent money, cell phone, he had clothes, everything in his backpack. They took it all*” (Porto Alegre)
	Q11	“*Poor people have fewer resources to recover from violence… if my cell phone gets stolen, replacing it would be more difficult compared to someone with more financial stability. So, I’m afraid for my safety*” (Porto Alegre)
Policy	Q12	“*When the police arrive like this, if you don’t run, you die, because they (the police) go out killing*” (São Paulo)
	Q13	“*I trust* [a drug trafficking group] *more than the police themselves, because if something goes down here,* [the drug trafficking group], *they'1l help me. If I wait for the police, I die and they don’t come*” (São Paulo)

### Physical risk environment

Violence in youth-frequented spaces created barriers to everyday tasks. In São Paulo, respondents anticipated that police raids would shut down nightlife. Many participants had anecdotes about running into alleys to evade brutal raids. One participant described a typical experience in which police officers wielded guns and had gas bombs [Q1]. Another participant recounted an experience of fleeing, as boys in the community threw bottles back at the police [Q2]. In Porto Alegre, the physical presence and actions of the law enforcement created a direct threat to individuals in these settings. One participant recounted a rending incident of unnecessary, direct police intervention at a friend’s house after a night out [Q3]. The use of a rifle and rough search tactics functioned as intimidation, targeting children. Such actions escalate tensions and contribute to a physically dangerous environment, even in situations that may not warrant such force. 

In neighborhoods along trafficking routes, the physical risk environment is compounded by limited freedom of movement due to pervasive community violence. Law enforcement and gang activity jointly alter the landscape, setting a precedent for expecting gunshots, aggravation, and death. One participant recounted a homicide in her neighborhood, in which a trafficking group killed a man on his sofa for his drug debts, in front of his mother [Q4]. Clients of drug traffickers bear the impact of violence, as do family members. The drug trafficking-associated violence extends beyond the reach of the trafficking groups themselves. Inter-group trafficking violence impacts the community at large, which poses a barrier to everyday tasks. One participant shared that their children had not been to school because of trafficking activity [Q5], illustrating that education is also affected by trafficking activity. Our analysis also discovered that residents perceive susceptibility to armed robberies. One account included a youth participant detailing how their father sought retaliation against a drug dealer that stole from them [Q6]. Robberies often entangle families in local trafficking dynamics and can escalate into further violence, including homicide.

### Social risk environment

A gendered pattern of risk emerged, as participants reported that violence and homicides predominantly targeted males. Moreover, we found that racialized risk exacerbates the social risk environment for Black youth. One participant identified their vulnerability to violence because of their Black identity [Q7]. Risks stemming from social position entrench marginalization by increasing youth’s exposure to dangerous situations, limiting mobility, and discouraging health-seeking behaviors. Gendered and racialized violence collectively exacerbates the social risk environment and may contribute to disparities in HIV transmission within these communities. 

Substance abuse as a response to community violence represents a key behavioral dimension within the HIV risk environment. In São Paulo, police raids on nightlife venues led to shifts in the social landscape of youth. As one participant explained, police surveillance intends to respond to drug use [Q8]. Police raids have created an environment where substance use and risky behaviors are both a response to and a consequence of violence. The disruption of traditional hubs for socializing has led youth to seek alternative spaces. Some participants noted that tobacco or cocaine use within their friend groups was central to their socializing, even if they personally did not use these substances. A few participants reported that they would go to downtown Porto Alegre to drink (an hour away driving) and would drink and drive. Some chose alternative venues where there are no raids, such as hookah bars, to gather. Police presence pushes behaviors such as substance use and risky sex to the margins, where drugs are readily available. Other youth have been pushed away from participating in the nightlife altogether [Q9], inspiring social isolation. Fear of violence restricts social activities, reducing healthy interactions, fostering anxiety, and concentrating on substance use as a coping mechanism, which further elevates HIV risk. 

### Economic risk environment

Community violence has kept youth from attending school, as families prioritize safety and classes are sometimes canceled for weeks due to shootings. Low economic resilience affects individuals in poorly resourced neighborhoods where community violence and instability are prevalent. Robberies were one pertinent type of economic risk; one participant reported that her mother had been robbed three times in Restinga. Another reported that her boyfriend had been robbed recently on his commute [Q10]. This encounter exemplifies a disruption of employment and livelihood. Robberies increased economic strain by forcing individuals that already face instability to rely on costly rideshares instead of public transport. Economic vulnerabilities in these areas are exacerbated by a lack of resources, making it difficult for residents to recover from material losses. The lack of financial stability means that losses from violence, such as theft, have a greater impact on people’s lives. 

### Policy risk environment

Despite free HIV testing and treatment, community violence poses an obstacle to accessing healthcare. Drug trafficking and shootings triggered safety protocols and violence alert systems, which participants noted had become increasingly common. Such measures frequently interrupted services and delayed care, as both residents and providers were confined indoors until the threat passed. Law enforcement plays a critical role in shaping the policy risk environment, especially in communities with high levels of violence, yet it often exacerbates the very issues it aims to mitigate. Community protection, human rights laws, and drug possession laws create a contentious dynamic. Participants described intentionally aggressive police interventions that escalated fear and violence, contributing to a sense of State-sanctioned danger [Q12]. Physical aggression and property manipulation were common, affecting both individuals engaged in drug trade and those outside of it. Some expressed a preference for gang protection over police intervention [Q13]. Gangs can promise locals protection in exchange for secrecy regarding their operations, leading some community members to feel safer under gang control, which is influenced by the aggressive nature of law enforcement and fosters distrust towards it. Aggressive policing practices have broader implications for the community’s social fabric and overall safety, operating as a policy factor that enhances factors contributing to the HIV risk environment. 

## Discussion

Our study examined community violence in HIV risk environments in the cities of São Paulo and Porto Alegre. We found pathways linking violence to HIV vulnerability across risk environment types. In the physical environment, police raids and trafficking disputes limited mobility and exposed youth to direct harm. In the social environment, racialized and gendered violence, coupled with disrupted nightlife and substance use, concentrated risky behaviors in unsafe spaces and reinforced stress, anxiety, and maladaptive coping. In the economic environment, robberies, income loss, and school disruptions strained livelihoods and magnified poverty’s impact. In the policy environment, aggressive policing and weak protections fostered fear and distrust, creating openings for trafficking groups to exert control and entrenching cycles of violence. This study is among the first to document how community violence shapes the HIV risk environment for Brazilian youth.

In the physical risk environment, we found that police raids and trafficking disputes limited youth mobility and exposed them to direct harm. These findings align with prior research showing that prolonged exposure to violence is linked to higher rates of depression and anxiety among Brazilian youth ^31^, which is linked to poor health promotion and risk HIV health behavior ^32^ ^,^ ^33^ ^,^ ^34^. The trauma of witnessing or being a victim of violence can lead to maladaptive coping mechanisms such as substance use and risky sexual behaviors, elevating the risk of HIV transmission. Frequent police raids and the availability of unregulated drugs drive youth to alternative, often riskier, social settings or stop them from socializing altogether. Alcohol and tobacco use have been linked to high-risk sexual behaviors among youth in Brazil ^35^; we reveal that community violence is one social factor connecting these behaviors. Thus, pervasive violence directly and indirectly heightens HIV risk among youth, establishing and perpetuating inequity.

The social risk environment reflects racialized and gendered risk. Our observations of racialized vulnerability are consistent with broader patterns indicating that Black youth are disproportionately targeted by police violence ^16^ ^,^ ^36^, as well as experiencing high HIV/AIDS mortality, which may signal inequities along the entire HIV prevention and care continuum ^28^. This process might be a fundamental cause of disparities in HIV incidence. Moreover, the gendered nature of violence in Brazil further exacerbates these issues. Males are disproportionately targeted by police brutality and gang violence, resulting in increased physical harm and social insecurity. Homicides particularly affect youth, with young men being the most frequent victims ^37^. Pervasive violence against men likely shapes broader social norms related to violence and masculinity, in which the prevalence of violence among young men reinforces the tolerance or valorization of aggression, contributing to cycles of violence ^37^ ^,^ ^38^. Within these contexts, young men may engage in risky behaviors such as joining gangs, perpetrating violence against women, or participating in the drug trade, further perpetuating the cycle of violence and increasing the risk of HIV transmission.

Community violence exacerbates the economic risk of the HIV risk environment for youth via robberies and interrupted school attendance. An increase in robberies was noted, which is in line with studies that identify aggravated burglary as one of the most frequent types of crime committed in Latin America ^37^. This is further supported by a cohort study of children born in the southern region of Brazil that revealed that 42% of 3,701 cohort members reported being victims of robbery by age 30 ^39^. Robberies threaten a downward spiral of social cohesion ^40^ and exacerbate material need ^41^, contributing to economic instability. School closures due to conflicts between trafficking groups and law enforcement further hinder economic development. Disruption of education due to shootings not only affects immediate learning opportunities but also has long-term economic implications for financial self-sufficiency. Then, the challenge of accessing stable employment forces individuals into high-risk activities to meet their basic needs, thereby accelerating economic instability. The literature suggests that in environments of sustained economic hardship and violence, informal economies (including trafficking) often prosper ^42^ ^,^ ^43^ ^,^ ^44^. While these economies can provide an income for individuals with limited opportunities, they also perpetuate the very issues they seek to mitigate, further entrenching communities into poverty and violence cycles. Consequently, individuals in these environments are more vulnerable to various risks, including HIV. Expanding economic opportunities and reducing dependence on illicit markets are therefore essential to improving health outcomes.

The policy risk environment is marked by a contradiction, while HIV testing and treatment are available with no charge, community violence rooted in organized crime and inadequate policy responses still hinder access. Within service delivery, frequent and unpredictable episodes of violence disrupt care, a challenge also documented among community health workers in Rio de Janeiro ^45^, where it contributes to staff turnover as workers leave for safer areas ^46^. Beyond service delivery, broader urban and policing dynamics further shape the policy risk environment. Intense urbanization has produced peripheral neighborhoods with limited infrastructure, public goods, and scarce State presence. Aggressive policing in these areas fosters fear and distrust, driving some residents to seek protection from trafficking groups that impose informal justice systems ^44^ ^,^ ^47^. “Tough on crime” policies and high levels of police violence and repression have shown themselves to be ineffective in preventing or reducing drug trafficking or substance use ^44^, instead perpetuating cycles of violence and counter-violence. Government strategies have combined increased policing with social initiatives to address community violence ^48^, yet scaling up policing has led to more reports that abusive acts perpetrated by law enforcement agents are frequent, even among new police cohorts ^49^. A large-scale reassessment of policing systems is urgent. Strengthening accountability and addressing the socioeconomic roots of gang formation are essential to reduce violence, improve public safety, and create more resilient communities. Policies need to prioritize community engagement, respect for human rights, and accountability to address both public security and public health concerns effectively.

The strengths of this study include applying the HIV risk environment framework to youth perspectives in two underexplored Brazilian communities and incorporating participant narratives to capture the lived experience of community violence. While our data do not directly connect to HIV outcomes because we focused on an at-risk population, the framework guides integration of our findings within established pathways to HIV vulnerability. Translation during analysis may have led to some loss of meaning, but this risk was mitigated via back-translating and cross-checking by bilingual authors. We did not systematically collect data on transgender identity or sexual orientation; future studies should consider how homophobia and transphobia operate alongside violence in shaping the HIV risk environment. Finally, as a community-based qualitative study interrupted by COVID-19, findings may not have reached full saturation and are not designed for broad transferability.

## Conclusion

We provide evidence of how community violence shapes the HIV risk environment in South and Southeast Brazil. This research contributes to a growing body of evidence that efforts aimed at reducing HIV risk must be multilevel, enhancing resilience at the community level while addressing both immediate circumstances of individuals and overarching policy structures that influence these environments. By highlighting experiences of police brutality, gang activity, and robberies, our study demonstrates how community violence acts both as a product and a determinant of environmental risk factors that accelerate HIV transmission among youth. Among solutions we suggest the development of programs to promote engagement in care, understanding that community violence is a barrier to access, legislation and assessment of policies to mitigate drug trafficking and police brutality, and economic empowerment of peripheral neighborhoods. Addressing community violence is essential for preventing HIV disparities among youth, as it reproduces inequity and remains a fundamental cause of health disparities.

## Data Availability

The research data are available upon request to the corresponding author.
